# One-stop procedure for persistent atrial fibrillation with cor triatriatum sinister under intracardiac echocardiography guidance: a case report

**DOI:** 10.3389/fcvm.2025.1650461

**Published:** 2025-11-07

**Authors:** Hong Cai Zhang, Bing xia Yang, Qian Nie, Jue Zhao, Ming Jun Han, De Lai Zhang, Wen Xie

**Affiliations:** 1 Department of Cardiology, Hospital of Chengdu University of Traditional Chinese Medicine, Chengdu, China; 2 Department of Cardiology, Chengdu University of Traditional Chinese Medicine, Chengdu, China

**Keywords:** cor triatriatum sinister, atrial fibrillation, one-stop procedure, radiofrequency ablation, left atrial appendage closure, ICE guidance

## Abstract

This case report describes the treatment of a 76-year-old male patient diagnosed with persistent atrial fibrillation (AF) and cor triatriatum sinister (CTS). The patient presented with palpitations and shortness of breath for four years, exacerbated over two weeks. Cardiac ultrasound and computed tomography angiography (CTA) confirmed Bank II type complete CTS, with all four pulmonary veins draining into an accessory atrium. The patient also had lacunar stroke and heart failure, with a CHA2DS2-VASc score of 5 and HAS-BLED score of 2, indicating high stroke risk and moderate bleeding risk. Given the anatomical abnormalities and clinical characteristics, we performed a one-stop procedure under intracardiac echocardiography (ICE) guidance, combining AF radiofrequency ablation and left atrial appendage closure (LAAC). Post-procedure recovery was uneventful, and follow-up transesophageal echocardiography showed no residual shunt or thrombus around the occluder. An antithrombotic regimen of rivaroxaban 15 mg once daily for three months followed by aspirin 100 mg once daily long-term was prescribed. This case highlights the critical role of ICE technology in complex cardiac anatomy and the importance of personalized antithrombotic strategies in high-risk AF patients.

## 1 Introduction

Cor triatriatum is a rare congenital cardiac anomaly accounting for approximately 0.1%–0.4% of all congenital heart diseases (1). Among them, cor triatriatum sinister (CTS) is the most common, classified as Bank I (partial) and Bank II (complete) (2). Complete CTS refers to all four pulmonary veins draining into an accessory atrium, while the presence or absence of communication between the true and accessory atria further divides it into subtypes A (communicating) and B (non-communicating). Our patient had Bank II-A subtype, characterized by complete and communicating features. AF is the most common sustained arrhythmia clinically, and managing AF in patients with congenital heart disease is more complex. For patients at high stroke risk (CHA2DS2-VASc ≥ 1) and higher bleeding risk (HAS-BLED ≥ 3), guidelines recommend novel oral anticoagulants (NOACs) for stroke prevention. However, for those with contraindications to anticoagulation or extremely high bleeding risk, LAAC is an effective alternative. In this case, due to advanced age, high stroke risk, and moderate bleeding risk, we opted for an ICE-guided one-stop procedure combining AF ablation and LAAC.

## 2 Case presentation

### 2.1 Patient information

A 76-year-old male presented with palpitations and shortness of breath for four years, exacerbated over two weeks. He had a history of lacunar stroke and heart failure for one year. On admission, his vital signs were stable, blood pressure was 120/75 mmHg, heart rate was 143 bpm, and he had irregularly irregular pulse without murmurs. Neurological examination was unremarkable. Complete blood count (CBC), liver and kidney function tests, coagulation profile, BNP, and troponin I showed no significant abnormalities.

### 2.2 Electrocardiogram (ECG) findings

**Figure 1:**
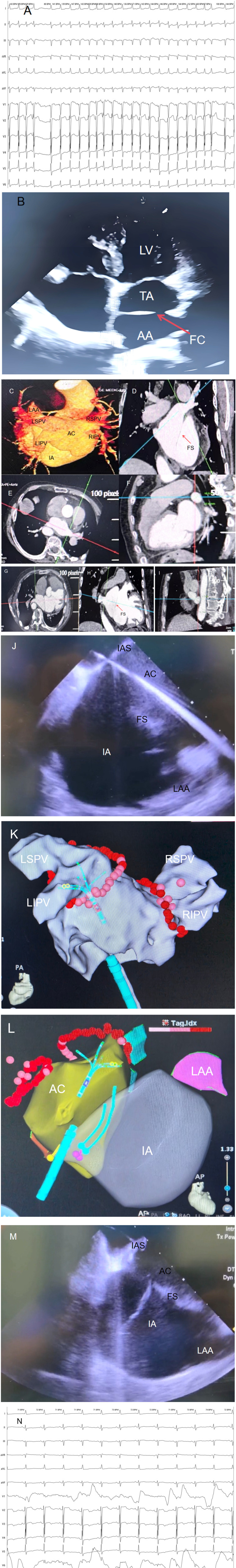
**(A)** Preoperative electrocardiogram (ECG) of the patient showing atrial fibrillation. **(B)** Transthoracic echocardiography (TTE) clearly visualizes the fibromuscular septum (FS), Intrinsic Atrium (IA), and Accessory Atrial Chamber (AC) in the patient. **(C–I)** Contrast-enhanced cardiac computed tomography (CE-CT) and 3D reconstruction of the patient, demonstrating the structural characteristics of cor triatriatum from multiple angles. The Accessory Atrial Chamber (AC) is connected to all four pulmonary veins (PVs), while the Intrinsic Atrium (IA) is connected to the left atrial appendage (LAA). **(J)** Intracardiac Echocardiography (ICE)-guided transseptal puncture to access the Accessory Atrial Chamber (AC). **(K)** Pulmonary vein antrum isolation (PVI) performed under guidance of the Johnson & Johnson Cato 3D mapping system. **(L)** Intracardiac Echocardiography (ICE) modeling illustrating the anatomical relationships: the Accessory Atrial Chamber (AC) is connected to all four pulmonary veins (PVs), and the Intrinsic Atrium (IA) is connected to the left atrial appendage (LAA). **(M)** Intracardiac Echocardiography (ICE)-guided catheter traversal across the fibromuscular septum (FS) into the Intrinsic Atrium (IA). **(N)** Following pulmonary vein isolation (PVI), cardioversion with a 150-J shock was performed, converting the patient to sinus rhythm with a heart rate of 71 beats per minute (bpm). IA, intrinsic atrium; AC, accessory atrial chamber; FS, fibromuscular septum; IAS, interatrial septum; LIPV, left inferior pulmonary vein; RIPV, right inferior pulmonary vein; LSPV, left superior pulmonary vein; RSPV, right superior pulmonary vein; ICE, intracardiac echocardiography; TTE, transthoracic echocardiography; CE-CT, contrast-enhanced cardiac computed tomography; PVI, pulmonary vein antrum isolation; LAA, left atrial appendage.

The preoperative ECG showed persistent AF with a ventricular rate of approximately 143 bpm, disappearance of P waves replaced by f waves, and absolutely irregular RR intervals; Electrocardiogram (ECG) revealed left axis deviation, ST-segment depression in multiple leads, and T-wave flattening with inversion (Figure 1A).

### 2.3 Imaging studies

Cardiac ultrasound findings:

1. A septum-like echo within the left atrium dividing it into a true atrium and an accessory atrium.
2. Enlarged left and right atria, right ventricle, and pulmonary hypertension (systolic pressure 48 mmHg).
3. Dilated aortic sinus and ascending aorta.
4. Reduced left ventricular diastolic function but normal systolic function (LVEF 65%).
5. Color Doppler showed that all four pulmonary veins drained directly into the accessory atrium and then communicated with the true atrium through the septal orifice (Figure 1B).

CTA findings:

1. A septum dividing the left atrium into an accessory and true atrium, with communication between them.
2. All four pulmonary veins drained into the accessory atrium, which communicated with the true atrium via the septal orifice.
3. No thrombus observed in the left atrial appendage (Figures 1C,I).
4. The size of the septal orifice was approximately 2.1 cm × 1.8 cm.

### 2.4 Preoperative assessment

Based on the CHA2DS2-VASc scoring system, the patient had congestive heart failure (1 point), age ≥75 years (1 point), hypertension (1 point), diabetes mellitus (1 point), and prior stroke (1 point), totaling 5 points, indicating high stroke risk. Based on the HAS-BLED scoring system, the patient scored 2 points for hypertension and age ≥65 years, indicating moderate bleeding risk. The patient expressed concern about the risk of bleeding associated with long-term anticoagulant use and declined long-term anticoagulant therapy. Due to his advanced age and comorbidities, a multidisciplinary team decided on an ICE-guided one-stop procedure combining AF ablation and LAAC to address both rhythm control and stroke prevention.

### 2.5 Surgical procedure

The surgery was performed under local anesthesia with preoperative anticoagulation using rivaroxaban. During the procedure, heparin was administered to maintain activated clotting time (ACT) between 300 and 350 s.

#### 2.5.1 AF radiofrequency ablation

Under ICE guidance, accurate puncture of the interatrial septum into the accessory atrium was achieved (Figure 1E). ICE confirmed the needle's position within the left atrium, followed by pulmonary vein isolation. Voltage mapping identified the electrical potentials around the pulmonary vein ostia, and circumferential pulmonary vein isolation was performed in the accessory atrium. Power settings were 45W for anterior walls and 50W for posterior walls, with saline irrigation at 17 ml/min and temperature maintained at 45°C. Lesion spacing did not exceed 5 mm, and pressure was kept between 5 and 15 g. Successful pulmonary vein isolation was achieved, followed by cardioversion to sinus rhythm (Figures 1K–N).

#### 2.5.2 Left atrial appendage closure

Under ICE guidance, the catheter was advanced through the septal orifice into the true atrium (Figure 1M). ICE confirmed the absence of thrombi in the left atrial appendage, which appeared well-formed. A disk-type occluder (LAmax-2436) was selected, positioned accurately, and deployed. Immediate post-procedural ICE confirmed proper placement of the occluder without pericardial effusion or residual shunt. Follow-up transesophageal echocardiography confirmed successful occlusion.

### 2.6 Postoperative management and follow-up

Postoperatively, the patient received rivaroxaban 15 mg once daily for three months. After three months, follow-up transesophageal echocardiography showed no residual shunt or thrombus, and therapy was switched to aspirin 100 mg once daily long-term. Currently, the patient has completed a 12-month follow-up, with good recovery, no chest pain or dyspnea, and continues to maintain sinus rhythm.

## 3 Discussion

### 3.1 Rarity, classification, and surgical challenges of CTS

CTS is a rare congenital anomaly, comprising 0.1%–0.4% of all congenital heart diseases (1). According to Bank classification, our patient had complete type II with communication (subtype A) (2). The dual classification method proposed by Zhu Xiaodong further categorized this as a “complex type”, associated with pulmonary hypertension and heart failure. Managing AF in such patients poses multiple challenges: abnormal left atrial anatomy, large communication orifices, and increased surgical risks due to advanced age and comorbidities. Thus, selecting appropriate imaging guidance and surgical strategy is crucial.

### 3.2 Role of ICE technology

In this case, ICE played a pivotal role:

1. Precise modeling: ICE combined with a three-dimensional electroanatomical mapping system created a detailed model of the left atrium, clearly delineating the accessory and true atria and their relationship with pulmonary veins and the septum (3).
2. Real-time navigation: ICE provided real-time visualization of catheter positions, reducing the risk of misplacement compared to traditional x-ray guidance. Studies show a 100% success rate for ICE-guided atrial septal puncture vs. 90% with x-ray (4).
3. Complication monitoring: ICE monitored for complications like pericardial effusion and pulmonary vein stenosis, none of which occurred in this case.
4. Reduced radiation exposure: ICE significantly reduced fluoroscopy time, lowering radiation exposure for both patient and operator (5).

### 3.3 Selection of antithrombotic regimen

For patients undergoing AF ablation and LAAC, antithrombotic management must balance stroke and bleeding risks. According to the latest 2025 AF stroke prevention guidelines, LAAC typically involves NOACs for 45 days followed by dual antiplatelet therapy (DAPT) for six months, then long-term single antiplatelet therapy (6). However, given the patient's age, heart failure, and prior stroke, we chose rivaroxaban 15 mg once daily for three months followed by aspirin 100 mg once daily long-term. Meta-analysis supports that short-term NOAC use post-LAAC reduces device-related thrombosis risk compared to DAPT (7).

### 3.4 The “one-stop” procedure

The “one-stop” procedure, as defined by He et al. (8), integrates atrial fibrillation (AF) ablation with left atrial appendage closure (LAAC) in a single session, minimizing procedural trauma and enhancing patient convenience. Its primary indications include drug-refractory AF, high stroke risk (CHA₂DS₂-VASc score ≥2), and bleeding-prone patients refusing long-term anticoagulation.

For patient selection:

- LAAC with occluders is preferred for those with favorable LAA anatomy (e.g., cauliflower or multilobular type) that matches device anchoring requirements, or who prioritize minimally invasive approaches.
- Surgical clipping/stapling combined with LAA excision suits patients with regular LAA morphology (e.g., chicken-wing type) amenable to mechanical closure, or those requiring concomitant cardiac procedures (e.g., mitral valve repair).
- Advantages and disadvantages: LAAC offers minimal invasiveness, faster recovery, and no need for general anesthesia (8), but requires strict anatomical compatibility and carries a small risk of residual shunt.

Surgical clipping boasts high success rates (>98%) but involves thoracotomy and prolonged rehabilitation.

Stapling ensures reliable LAA exclusion but demands advanced surgical expertise and is less tolerant of anatomical variations.

As emphasized by He et al. (8), the choice of procedure must be individualized—balancing anatomical features, patient preferences, and institutional proficiency—to optimize outcomes for complex AF management.

## 4 Conclusion

This case illustrates the essential role of ICE technology in managing complex cardiac anatomies and underscores the importance of individualized antithrombotic strategies in high-risk AF patients. An ICE-guided one-stop procedure combining AF ablation and LAAC offers a safe and effective treatment option for patients with persistent AF and CTS. Personalized antithrombotic regimens should be tailored to each patient's specific stroke and bleeding risk profiles to achieve optimal clinical outcomes.

The authors have read the CARE Checklist (2016) and prepared and revised the manuscript according to this guideline.

## Data Availability

The original contributions presented in the study are included in the article/Supplementary Material, further inquiries can be directed to the corresponding author.
